# Evolutionary applications research highlight for issue 1

**DOI:** 10.1111/eva.12238

**Published:** 2015-01-21

**Authors:** Britt Koskella

As we highlight each month in this section, the application of evolutionary theory to issues affecting the health and well-being of human, agricultural, and natural populations is gaining increasing momentum. In a recent review article written for *Science*, Scott Carroll et al. take on the now monumental task of synthesizing the many ways that evolutionary biology can be used to address global challenges (Carroll et al. 2014). They comprehensively explore the main problems being tackled with an evolutionary approach, ranging from populations evolving too quickly (such as emerging pathogens or pests evolving resistance to treatment) to populations not evolving quickly enough (for example those being negatively affected by human-mediated change).

The authors begin by identifying what they see as the two key paradigms of applied evolutionary biology: (i) managing contemporary evolution (i.e., manipulating the rapid evolutionary response of short-lived organisms with large population sizes, such as bacterial pathogens) and (ii) altering the phenotype–environment mismatch (i.e., responding to populations of long-lived organisms such as trees that are no longer well adapted to their local environment due to shifts in climatic conditions or changes in biotic interactions). As a great example of such a mismatch, the authors highlight the increasing rates of obesity, diabetes, and heart disease in the human populations as a result of a more sedentary lifestyle with diets rich in sugars and fat. They then identify a number of promising research avenues that either have addressed or have the potential to address current global challenges, covering a wide range of approaches including the use of genetic engineering to more appropriately match genomes to their environment, the use of ‘refuges’ in agriculture and combination treatments against pests and pathogens to hinder the evolution of resistance, and introducing nonlocal genotypes which are predicted to perform better under given environmental conditions into natural populations to increase local adaptation.

The article nicely separates these conceptual approaches into strategies for slowing unwanted evolution or directly influencing fitness of pests and pathogens, strategies for reducing the mismatch between phenotype and the local environment, and strategies for increasing group performance by selecting on group-level traits. For example, the authors discuss the success of artificially selecting for group yield in agricultural plots rather than individual fitness as a means for decreasing competition among plants. Critically, the piece also emphasizes the need to take a unified approach in meeting international objectives for sustainable development and suggests a need for stricter enforcement of guidelines in order to ensure best practice is achieved despite temptation to put profit or immediate success ahead of sustainable solutions.

Overall, the review acts as a unique and remarkable resource both for researchers and students who are new to the field of applied evolution and those who actively contribute to the field.
